# Effect of Ang II Receptor Inhibition on GSK-3β/CREB/BDNF Signalling in REM Sleep Deprivation-Induced Memory Impairment

**DOI:** 10.1007/s11064-025-04660-z

**Published:** 2026-01-14

**Authors:** Nazan Elma, Hale Sayan Özaçmak, İnci Turan

**Affiliations:** Department of Physiology, Faculty of Medicine, Zonguldak Bülent Ecevit University, Kozlu, 67600 Zonguldak, Turkey

**Keywords:** Cognitive neuroprotection, Energy metabolism, Oxidative stress modulation, Synaptic plasticity

## Abstract

REM (rapid eye movement) sleep deprivation causes serious impairments in hippocampus-dependent learning and memory. This study examined whether the angiotensin II receptor blocker telmisartan, given at two different doses, could reduce cognitive deficits and affect molecular pathways related to chronic REM sleep deprivation. Thirty-two male Wistar-Albino rats (200–280 g, 3 months old) were randomly divided into four groups (*n* = 8): control, sleep deprivation (SD), telmisartan-treated SD groups at 1 mg/kg (SD+Tel1) and 3 mg/kg (SD+Tel3). Chronic REM sleep deprivation was induced for 21 days using the modified multiple platform (MMP) method. Telmisartan or distilled water was administered orally once daily. Cognitive performance was tested in the Morris water maze, assessing escape latency and time spent in the target quadrant. After behavioral tests, hippocampal and prefrontal cortex samples were analyzed for brain-derived neurotrophic factor (BDNF), cAMP response element-binding protein (CREB), glycogen synthase kinase-3 beta (GSK-3β), monocarboxylate transporter 2 (MCT2), and lactate dehydrogenase (LDH) levels, while plasma samples were analyzed for corticosterone (CORT) levels. Brain levels of malondialdehyde (MDA), reduced glutathione (GSH), nitrate, and glycogen were also measured. Sleep-deprived rats showed impaired learning and memory with longer escape latency and reduced time spent of target quadrant. Telmisartan-treated SD groups demonstrated significantly improved cognitive performance, increased BDNF and CREB expression, decreased GSK-3β levels, and balanced oxidative stress markers. In conclusion, telmisartan protected against cognitive and biochemical damage caused by chronic REM sleep deprivation, likely through modulation of GSK-3β/CREB/BDNF signaling and reduction of oxidative stress.

## Introduction

REM sleep plays a crucial role in synaptic plasticity, learning, and memory consolidation [1, 2]. During this phase, the brain demonstrates intense neuronal activity, increased metabolism, and vivid dreaming, all of which contribute to emotional regulation and cognitive processing [3]. Chronic REM sleep deprivation (RSD) has been reported to impair hippocampus-dependent memory and may disrupt long-term potentiation, a key mechanism of learning and synaptic plasticity [4, 5]. However, the extent of these effects appears to vary depending on species, deprivation method, duration, and the specific hippocampal region examined, and they may also be partially influenced by stress-related confounds inherent to REM deprivation protocols [6] Prolonged RSD is suggested to induce molecular and structural alterations in the hippocampus, including synaptic dysfunction, altered neurotransmitter balance, and dysregulation of signalling pathways critical for neuroplasticity [5]. Stress-related responses, which are inherent to RSD paradigms, may also contribute to the observed findings. In addition, exposure to RSD before or after learning negatively affects acquisition and memory consolidation, respectively [7]. Given the growing prevalence of chronic RSD due to lifestyle factors, understanding the molecular mechanisms underlying RSD-induced cognitive deficits has become increasingly important [3, 8].

Moreover, chronic RSD is associated with oxidative stress, neuroinflammation, and neuronal loss, especially in the hippocampus [5, 8]. These effects are mediated by mitochondrial dysfunction, increased reactive oxygen species (ROS), and impaired antioxidant defense mechanisms [9]. Sleep deprivation is known to modulate hypothalamic–pituitary–adrenal (HPA) axis activity, often leading to transient or context-dependent alterations in corticosterone (CORT) secretion rather than uniform increases. While some experimental models report HPA axis activation and elevated CORT levels during RSD, this response is not universal and appears to vary across deprivation paradigms [6, 10].

Available evidence suggests that REM sleep loss can contribute to metabolic disturbances in hippocampal neurons, including decreased mitochondrial efficiency, increased ROS accumulation, and weakened activity-dependent signalling [11]. As both CORT-dependent and CORT-independent pathways are engaged under these conditions, the convergence of these mechanisms ultimately leads to CREB inactivation and subsequent downregulation of BDNF expression, a process further compounded by reduced neuronal activity during REM deprivation [11, 12].

Within this context, REM deprivation has consistently been shown to suppress hippocampal CREB phosphorylation and reduce BDNF expression, indicating that the CREB–BDNF axis represents one of the most robustly affected pathways in REM deprivation models. In parallel REM deprivation studies also demonstrate a marked increase in GSK-3β activity, linking REM loss to downstream tau-related pathology and mitochondrial vulnerability. Downregulation of BDNF and CREB contributes to synaptic failure and cognitive impairment, whereas excessive GSK-3β activation facilitates neuronal apoptosis and tau phosphorylation [4, 13] thereby exacerbating hippocampal vulnerability under chronic RSD conditions.

The renin–angiotensin system (RAS), beyond its classical cardiovascular role, is widely expressed in the central nervous system and modulates neuronal excitability, neuroinflammation, and synaptic transmission [14, 15]. Although REM-specific RAS activation in the hippocampus has not been extensively characterized, several studies demonstrate that sleep deprivation and stress states upregulate Ang II/AT1R signalling in central regions [16–18]. Given that Ang II suppresses hippocampal LTP and impairs memory, and that hippocampal RAS is highly responsive to oxidative and inflammatory stressors—which are prominent features of REM sleep deprivation—the involvement of RAS pathways under RSD conditions is biologically plausible and mechanistically supported.

Angiotensin II receptor blockers (ARBs), such as telmisartan, can cross the blood–brain barrier and exert neuroprotective effects through anti-inflammatory and antioxidant actions [19, 20]. Telmisartan is unique among ARBs because it also acts as a partial agonist of peroxisome proliferator-activated receptor-gamma (PPAR-γ), enhancing mitochondrial biogenesis, lipid metabolism, and cellular antioxidant capacity [21]. Recent findings suggest that ARB treatment can improve cognitive function by modulating intracellular pathways such as GSK-3β/CREB/BDNF, thereby supporting hippocampal plasticity [14]. Given that REM sleep deprivation imposes substantial metabolic stress on hippocampal neurons—characterized by impaired mitochondrial efficiency, increased ROS production, and disrupted lactate shuttling—the PPAR-γ–mediated actions of telmisartan provide a mechanistic advantage by enhancing mitochondrial biogenesis and restoring cellular redox balance [22]. Telmisartan’s high lipophilicity allows it to readily cross the blood–brain barrier, achieving pharmacologically relevant levels in hippocampal and cortical tissue even under conditions of stress-induced BBB alterations, a property that distinguishes it from less brain-penetrant ARBs. Moreover, telmisartan’s high brain permeability ensures effective AT1 receptor blockade within hippocampal and cortical regions, where sleep loss amplifies Ang II–driven oxidative and metabolic dysfunction, making it particularly suitable for counteracting RSD-induced neurometabolic deficits [23]. Taken together, these properties position telmisartan as a biologically plausible candidate for mitigating RSD-related hippocampal vulnerability [24].

Recent data show that astrocytic regulation of lactate availability and MCT2-dependent neuronal uptake plays a critical role in maintaining sleep–wake architecture, particularly through modulation of orexinergic activity in the lateral hypothalamus [25]. Although current evidence derives primarily from normal sleep–wake physiology rather than selective REM deprivation, the dynamic nature of astrocyte–neuron lactate shuttling suggests that REM sleep loss could plausibly disrupt this metabolic coupling.

Fluctuations in lactate dehydrogenase (LDH) levels are often interpreted as an indirect, albeit non-specific, proxy for alterations in glial or neuronal metabolic burden, reflecting potential adjustments in cellular energy demand or stress-related biochemical activity [26]. Given the high energetic requirements of neuronal networks during REM sleep, disruption of this sleep stage may impair lactate-mediated metabolic coupling between astrocytes and neurons. In this context, LDH was included in the present study not as a primary metabolic outcome, but as a secondary marker reflecting cellular stress associated with REM sleep deprivation–induced metabolic imbalance.

In addition, telmisartan influences multiple metabolic and stress-responsive pathways. Telmisartan regulates MCT2, a key transporter mediating the astrocyte-to-neuron lactate shuttle [27], and may modulate LDH activity, a downstream indicator of shifts in cellular energy metabolism [24]. By supporting mitochondrial function, enhancing anti-oxidative capacity, and stabilizing neuron–glia metabolic interactions—processes known to be sensitive to sleep deprivation— telmisartan’s combined AT1-receptor blockade and PPAR-γ activation may facilitate more efficient metabolic adaptation under RSD. Through these pleiotropic mechanisms, including improvements in cellular energy handling and stress resilience, telmisartan may act as both a neuroprotective and neurometabolic modulator, contributing to cellular resilience under conditions of sleep-loss-related stress.

Therefore, the present study aimed to investigate the potential neuroprotective effects of telmisartan, administered at two different doses, on spatial learning and memory impairments induced by chronic REM sleep deprivation in rats. Furthermore, we sought to elucidate the underlying molecular mechanisms by examining GSK-3β, CREB, BDNF, MCT2, and LDH levels in the hippocampus and prefrontal cortex, as well as plasma CORT concentrations. We hypothesized that telmisartan treatment would improve learning and memory performance and partially counteract the molecular alterations induced by REM sleep deprivation, thereby providing potential therapeutic relevance for sleep deprivation–related cognitive deficits.

Although REM sleep deprivation is known to impair cognitive performance and hippocampal plasticity, the contribution of the brain renin–angiotensin system to these alterations remains poorly understood. To date, no study has investigated the effects of angiotensin II receptor blockers on REM sleep deprivation–induced cognitive deficits. Moreover, the potential interaction between synaptic plasticity–related signalling pathways (GSK-3β/CREB/BDNF) and metabolic regulators such as MCT2 and LDH has not been examined within the same experimental framework. Importantly, telmisartan, which possesses both angiotensin II receptor–blocking and PPAR-γ–activating properties with high brain penetrance, has not previously been evaluated in a sleep deprivation paradigm. Therefore, the present study aimed to address these gaps by investigating the effects of telmisartan on behavioural performance and hippocampal molecular alterations following REM sleep deprivation.

## Methods

### Animals

A total of thirty-two adult male Wistar-Albino rats (200–280 g) were utilized in this study in order to minimize hormonal variability and the well-documented sex-dependent differences in sleep architecture, circadian regulation, EEG responses, and rebound mechanisms following sleep deprivation in rodents [28]. All animals were obtained from the Experimental Research Application and Research Center at Zonguldak Bülent Ecevit University, Turkey. The animals were acclimated while being group-housed (eight rats per cage for each experimental group) and maintained under controlled laboratory conditions, including a 12-hour light/dark cycle and a stable ambient temperature of 23 ± 2 °C. Standard rodent chow and water were provided ad libitum throughout the experimental period. Behavioral experiment was scheduled between 12:00 PM and 14:00 PM to minimize potential circadian effects.

### Experimental Design

**Fig. 1:**
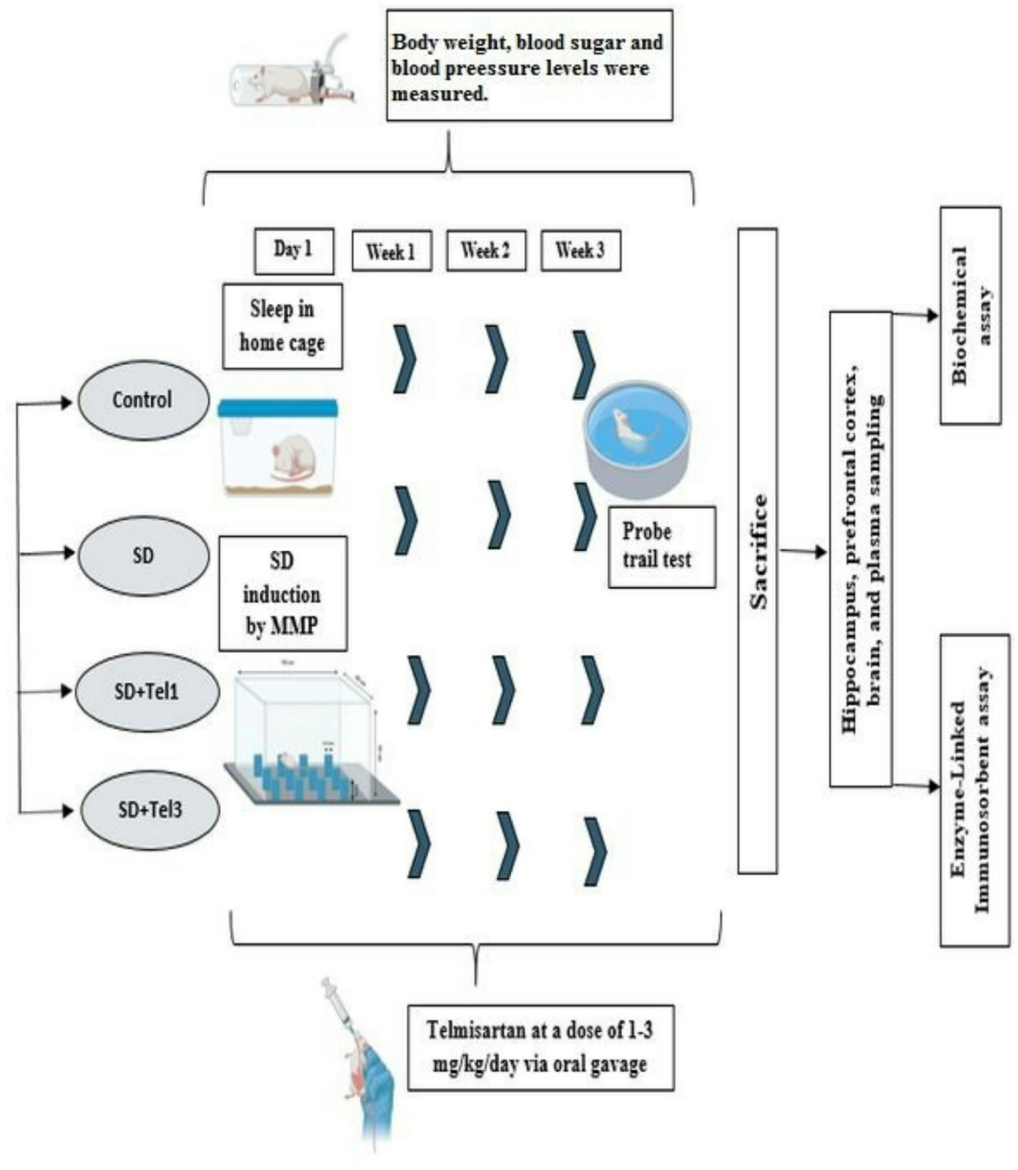
Graphical summary of the study design illustrating the protective effects of telmisartan against REM sleep deprivation–induced impairments. Figure created with publication rights from BioRender.com

The experimental design is illustrated in Fig. 1. To evaluate the effects of telmisartan on learning and memory deficits induced by chronic REM sleep deprivation, 32 adult male Wistar-Albino rats were randomly assigned to four groups (*n* = 8 per group) using a computer-generated randomization list:

1. Control group, maintained in standard home cages and administered distilled water (vehicle) once daily by oral gavage;
2. SD group, subjected to chronic REM sleep deprivation for 21 days using the MMP method and given distilled water (vehicle) once daily by oral gavage in the same volume as drug-treated groups;
3. SD + Tel1 group, exposed to chronic REM sleep deprivation for 21 days and administered telmisartan dissolved in distilled water at a dose of 1 mg/kg/day via oral gavage; and.
4. SD + Tel3 group, subjected to REM sleep deprivation for 21 days and treated with telmisartan dissolved in distilled water at 3 mg/kg/day via oral gavage [29].

All gavage procedures and volumes were identical across groups to control for vehicle and handling effects.

For sleep deprivation, the animals were placed on the platforms daily from 16:00 PM until the following day at 10:00 AM [12]. Telmisartan was administered daily at 10:00 AM [30], immediately following the REM sleep deprivation period, to maintain consistent pharmacokinetic and physiological conditions across all animals. The Morris Water Maze (MWM) behavioral tests were conducted during the final six days of the 21-day treatment, with animals returned to their home cages for rest periods after each training session to reduce potential stress-learning interactions.

After the behavioral tests, the rats were sacrificed under ketamine (60 mg/kg) and xylazine (10 mg/kg) anesthesia by cardiac puncture, and the collected blood was processed for plasma preparation. Whole brains were removed, weighed, and placed on ice. The hippocampus and prefrontal cortex were rapidly dissected and stored at − 80 °C for ELISA assays. After separation of the cerebellum and brainstem, the remaining brain tissue was also stored at − 80 °C until further biochemical analyses, including measurements of MDA, GSH, nitrate, and glycogen levels. Behavioral testing and biochemical/ELISA procedures were carried out according to standardized protocols, and outcome assessments were performed by personnel who were not involved in group assignment.

### Measurement of Body Weight

In our study, body weight was measured four times in total — on the first day, at the end of the first week, the second week, and the third week.

### Measurement of Blood Glucose Levels

For blood glucose measurements, blood samples were collected from the lateral tail vein of the animals. Initially, the tails were cleaned with 70% ethyl alcohol. To facilitate blood pooling, gentle pressure was applied to the proximal part of the tail, and a drop of blood was obtained by puncturing the vessel with an 18G needle. The blood drop was then placed onto a test strip of a calibrated eBsensor^®^ device for glucose measurement. Blood glucose levels were measured because chronic stress–related interventions can alter glucose homeostasis, and telmisartan is known to improve insulin sensitivity and metabolic regulation [31]. Measurements were performed four times in total — on the first day of the experiment and at the end of the first, second, and third weeks — between 10:00 and 12:00, a time window selected to minimize circadian-driven variability in glucose levels [32].

### Measurement of Blood Pressure

In our study, trial measurements were conducted during the week prior to the experiment to acclimate the animals to the blood pressure measurement procedure. Blood pressure was measured from the tail using the PowerLab system in conjunction with LabChart 7 Pro. Measurements were performed four times in total — on the first day of the experiment, and at the end of the first, second, and third weeks, between 10:00 and 12:00. Each measurement session lasted 5 min, with recordings taken at 1-minute intervals. The average of these five values was used for analysis [33].

### Measurement of Body Temperature

Body temperature measurements were performed under brief anesthesia to minimize handling-related stress. Rats were anesthetized with ketamine (60 mg/kg) and xylazine (10 mg/kg) and placed in the supine position on days 1 and 21 of the experimental protocol. Body temperature was recorded immediately after anesthesia induction via rectal probe using a Harvard Apparatus Homeothermic Monitor. Measurements were performed sequentially for all animals in each group between 17:00 and 20:00, accounting for the time required to complete the procedure for each rat [34]. Although anesthetic agents may influence thermoregulation and metabolic rate, the same anesthesia protocol was applied uniformly across all experimental groups, thereby minimizing potential systematic bias.

### Sleep Deprivation Protocol

Chronic REM sleep deprivation was produced using a modified version of the MMP method [35]. The sleep-deprived rats were housed in a tank (90 × 50 × 50 cm) containing several small circular platforms (6.5 cm in diameter) for a period of 21 days. Each platform was elevated approximately 2 cm above the water surface. This approach is based on the phenomenon that rats experience a loss of muscle tone upon entering REM sleep, which causes them to fall into the water and awaken [35]. The platforms were spaced about 10 cm apart, allowing the animals to move freely from one to another. No large-platform control (LPC) or stress-control group was included; comparisons were made solely against home-cage control animals.

Throughout the deprivation period, the rats were kept on the platforms daily from 16:00 to 10:00 the following morning, with unrestricted access to food and water, and eight animals were accommodated together in each tank. Following each deprivation phase, the animals were returned to their home cages for a 6-hour sleep opportunity from 10:00 to 16:00. This 16:00–10:00 interval was selected because it corresponds to the peak period of paradoxical (REM) sleep in rats [36]. The RSD protocol was applied for a total of 21 days. During this period, the water in the tanks was replaced once daily at the same time, while the animals rested in their home cages. Control animals, on the other hand, were maintained in their home cages and permitted normal, undisturbed sleep.

### Morris Water Maze (MWM) Test

Spatial learning and memory abilities of all subjects were evaluated using the Morris Water Maze (MWM) test [37]. The apparatus consisted of a circular pool (150 cm in diameter, 60 cm deep) filled with opaque water maintained at 22 ± 1 °C. A submerged escape platform (10 cm in diameter) was placed approximately 2 cm below the water surface. Stable visual cues were positioned on the surrounding walls to facilitate spatial orientation during the trials.

On the first day, the rats were introduced to the task with a visible platform. During the subsequent four consecutive training days, the pool was divided into four quadrants (north, east, south, and west), and each animal was released from different starting points to locate the hidden platform within 60 s (measured as escape latency). Each rat completed four acquisition trials per day, with inter-trial intervals of 30–60 s. Animals unable to find the platform within the allotted time were gently directed to it and allowed to remain there for 15 s.

Behavioral assessments in the MWM were carried out during the final six days of the sleep deprivation protocol. The visible platform trial was conducted on the first day, followed by four days of hidden platform training with escape latency recorded each day. After each daily MWM session, the animals were placed back into their home cages until 16:00, providing a brief standardized rest period before the REM deprivation procedure resumed. On the sixth day, a probe trial was administered in which the platform was removed, and the duration spent by each rat in the target quadrant was measured to assess spatial memory retention. Manual scoring was performed independently by two trained observers to minimize observer bias.

### Biochemical Analysis

MDA, GSH, glycogen and nitrate levels were measured in brain (after dissection of the PFC, hippocampus, cerebellum, and brainstem) tissue. Brain tissues were homogenized and centrifuged, and MDA content was determined using the thiobarbituric acid (TBA) assay, with absorbance read at 535 nm [38]. GSH levels were measured spectrophotometrically using 5,5’-dithiobis-2-nitrobenzoic acid (DTNB) [39]. Brain glycogen was quantified by homogenization in KOH, ethanol precipitation, and reaction with phenol and H2SO4, with absorbance measured at 490 nm [40]. Nitrate concentrations were determined in brain homogenates by reaction with vanadium chloride, sulfanilamide, and N-(1-naphthyl)ethylenediamine dihydrochloride (NEDD), and absorbance was read at 540 nm [41].

### ELISA Assays

Enzyme-linked immunosorbent assays (ELISA) were performed to measure BDNF (cat # 201-11-0477), CREB (cat # 201-11-0040), GSK-3β (cat # 201-11-2686), MCT-2 (cat # 201-11-6119), and LDH (cat # 201-11-0531) levels in the bilateral hippocampus and PFC using commercially available rat ELISA kits (SunRed Biotechnology, China) according to the manufacturer’s instructions. Plasma CORT levels were measured using the Rat CORT ELISA kit (cat # 201-11-0497). All tissue and plasma samples were processed and analyzed following the same standardized protocol. All assays were performed using kits from a single lot to minimize batch-to-batch variability. Each sample was run in duplicate, and all analytes were measured on a single plate to avoid inter-plate variability. Appropriate dilution factors were applied to ensure readings within the linear range of the standard curve.

### Statistical Analysis

Statistical analyses were performed using Jamovi software, version 2.3 (Jamovi Project, Sydney, Australia). Group comparisons for numerical variables were conducted using the Kruskal–Wallis test, with post hoc Dunn’s test for pairwise subgroup comparisons with Bonferroni correction when significant differences were detected). Repeated measures were evaluated using the Friedman test, as appropriate; significant Friedman results were further analyzed with Dunn’s post hoc test with Bonferroni correction. Normality and homogeneity were assessed using Shapiro–Wilk test, and because several variables did not meet these assumptions, nonparametric procedures were applied. Effect sizes were reported as epsilon squared (ε²) for Kruskal–Wallis analyses and Kendall’s W for Friedman tests. All these tests were performed using Jamovi’s built-in nonparametric test modules. Data are presented as median (minimum–maximum), and p-values < 0.05 were considered statistically significant.

## Results

### Body Weight and Blood Glucose Levels

Weekly body weight changes differed among the groups, as shown in Table 1. The Control group showed progressive weight gain over the study period (χ²(3) = 16.27, *p* < 0.001, W = 0.68) whereas the SD group exhibited a consistent reduction in body weight (χ²(3) = 16.27, *p* < 0.001, W = 0.68). Body weight was not restored by telmisartan treatment (*p* > 0.05). Blood glucose levels were generally comparable among groups, although a notable reduction from baseline was observed in the SD + Tel1 group at week 3 (χ²(3) = 13.93, *p* = 0.003, W = 0.58) also illustrated in Table 1.

**Table 1:** Effect of REM sleep deprivation and telmisartan treatment on body weight and blood glucose levels in rats

Groups	Body Weight				Blood Glucose Levels			
	Day 1	Week 1	Week 2	Week 3	Day 1	Week 1	Week 2	Week 3
Control	263 ± 11	269 ± 11	292 ± 12^a^	304 ± 11^a, b^	127 ± 11	111 ± 6	98 ± 8	97 ± 8
SD	252 ± 4	238 ± 4^a^	237 ± 3^a^	234 ± 2^a^	114 ± 4	113 ± 6	96 ± 11	109 ± 8
SD + Tel1	268 ± 8	248 ± 7	241 ± 6^a^	243 ± 5	263 ± 7	263 ± 5	263 ± 6	263 ± 3^a^
SD + Tel3	228 ± 9	214 ± 8	204 ± 8^a^	210 ± 9^a^	98 ± 5	103 ± 8	105 ± 9	99 ± 7

Values are presented as mean ± SEM, *n* = 8 per group

Repeated measures were evaluated using the Friedman test, as appropriate; significant Friedman results were further analyzed with Dunn’s post hoc test. a: *p* < 0.05, different from day 1; b: *p* < 0.05, different from week 1

### Body Temperature and Blood Pressure Levels

Weekly body temperature levels differed among the groups, as shown in Table 2. Compared with the Control group, body temperature was significantly reduced in both the SD + Tel1 and SD + Tel3 groups (H(3) = 23.62, *p* < 0.001, ε² = 0.74). In addition, the SD + Tel3 group showed a significant decrease in body temperature compared with the SD group (H(3) = 23.62, *p* < 0.001, ε² = 0.74). Blood pressure levels were similar among groups on day 1, at week 2, and at week 3. At week 1, however, blood pressure increased in the SD group, whereas it was reduced in the SD + Tel3 group due to the effect of Telmisartan (χ²(3) = 16.27, *p* < 0.001, W = 0.68) (Table 2).

**Table 2:** Effect of REM sleep deprivation and telmisartan treatment on blood pressure levels in rats

Groups	Body Temperature		Blood Pressure Levels				
	Day 1	Week 3	Day 1	Week 1	Week 2		Week 3
Control	37.0	36.5 ± 0.2	76 ± 4	74 ± 5	70 ± 3		74 ± 2
SD	37.0	35.9 ± 0.2	70 ± 4	94 ± 2*	70 ± 5		72 ± 6
SD + Tel1	37.0	35.2 ± 0.2*	78 ± 4	80 ± 3	70 ± 2		81 ± 4
SD + Tel3	37.0	34.6 ± 0.1*,#	74 ± 4	65 ± 2^#^	73 ± 5		69 ± 4

Values are presented as mean ± SEM, *n* = 8 per group

Repeated measures were evaluated using the Friedman test, as appropriate; significant Friedman results were further analyzed with Dunn’s post hoc test. *p < 0.05, different from control group #*p* < 0.05, different from SD group

### REM Sleep Deprivation–Related Alterations in Spatial Learning and Memory: Protective Role of Telmisartan

**Fig. 2:**
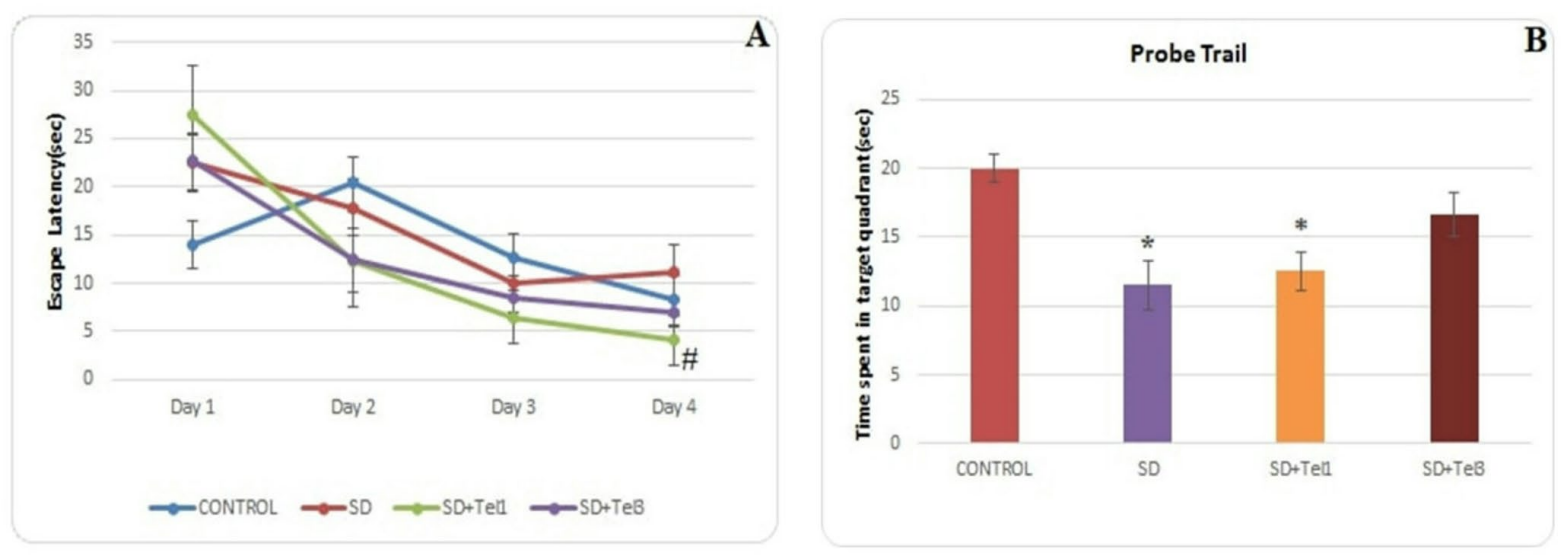
Effect of telmisartan on learning and memory performance of rats tested in the Morris water maze. **A** Escape latency of rats in the hidden platform test for four consecutive days. **B** Time spent in the target quadrant after 21 days of REM sleep deprivation. Repeated measures were evaluated using the Wilcoxon signed-rank or Friedman test, as appropriate; significant Friedman results were further analyzed with Dunn’s post hoc test. The data are expressed as median (min-max). *p < 0.05, significantly different from control group; # *p* < 0.05, significantly different from SD group

All rats were trained in the MWM until they reached a comparable level of acquisition performance. Escape latency were similar among the groups during the first three days of training (Fig. 2A). On day 4, SD + Tel1 rats reached the platform faster than the SD group, indicating an improvement in spatial learning with Telmisartan treatment (H(3) = 10.29, *p* = 0.010, ε² = 0.26). Memory performance was evaluated in the probe trial by measuring the time spent in the target quadrant. Compared with the Control group, both SD (H(3) = 11.46, *p* = 0.029, ε² = 0.30) and SD + Tel1 (H(3) = 11.46, *p* = 0.049, ε² = 0.30) rats spent less time in the target quadrant, reflecting memory impairment induced by RSD (Fig. 2B).

### REM Sleep Deprivation–Induced Alterations in Oxidative and Metabolic Markers: Modulatory Role of Telmisartan

To examine the impact of REM sleep deprivation and telmisartan administration on oxidative and metabolic markers, MDA, GSH, nitrate, and glycogen levels were measured in brain (after dissection of the PFC, hippocampus, cerebellum, and brainstem). As presented in Table 3, brain MDA was significantly reduced in the SD + Tel3 group compared with SD (H(3) = 8.86, *p* = 0.031, ε² = 0.21). Brain GSH content was elevated in SD + Tel3 animals compared with Control (H(3) = 24.55, *p* < 0.001, ε² = 0.79) and SD (H(3) = 24.55, *p* = 0.001, ε² = 0.79). Furthermore, brain nitrate levels were higher in SD (H(3) = 13.93, *p* = 0.008, ε² = 0.39) and SD + Tel3 (H(3) = 13.93, *p* = 0.007, ε² = 0.39) groups compared with Control, and glycogen content was elevated in SD + Tel1 (H(3) = 7.96, *p* = 0.038, ε² = 0.18). compared with SD. These findings suggest that telmisartan, particularly at higher doses, can attenuate oxidative stress and modulate energy metabolism under REM sleep deprivation (Table 3).

**Table 3:** Effect of REM sleep deprivation and Telmisartan treatment on oxidative and metabolic markers in rat brain tissue

	Control	SD	SD + Tel1	SD + Tel3
Brain MDA	145.07 (100.69–222.72)	168.53 (104.11–252.59)	171.09 (62.29–228.69)	69.12 (47.79–193.71)^#^
Brain GSH	9.09 (8.21–10.89)	9.71 (8.69–10.91)	11.31 (9.45–11.82)	12.71 (12.07–13.79)*^,#^
Brain Nitrate	7.29 (3.50–10.75)	15.18 (6.95–32.35)*	11.48 (10.00–14.05)	14.21 (5.50–18.10)*
Brain Glycogen	8.82 (5.93–10.08)	6.66 (5.17–8.40)	8.62 (7.66–10.34)^#^	7.21 (6.29–9.71)

Values are presented as median (min–max), *n* = 8 per group

Group comparisons for numerical variables were conducted using the Kruskal–Wallis test (with post hoc Dunn’s test for pairwise subgroup comparisons when significant differences were detected) *p < 0.05, different from control group # *p* < 0.05, different from SD group & *p* < 0.05, different from SD + Tel1 group

### Influence of REM Sleep Deprivation and Telmisartan Administration on Hippocampal and Prefrontal Cortex Expression of BDNF, CREB, GSK-3β, MCT2, and LDH

**Fig. 3:**
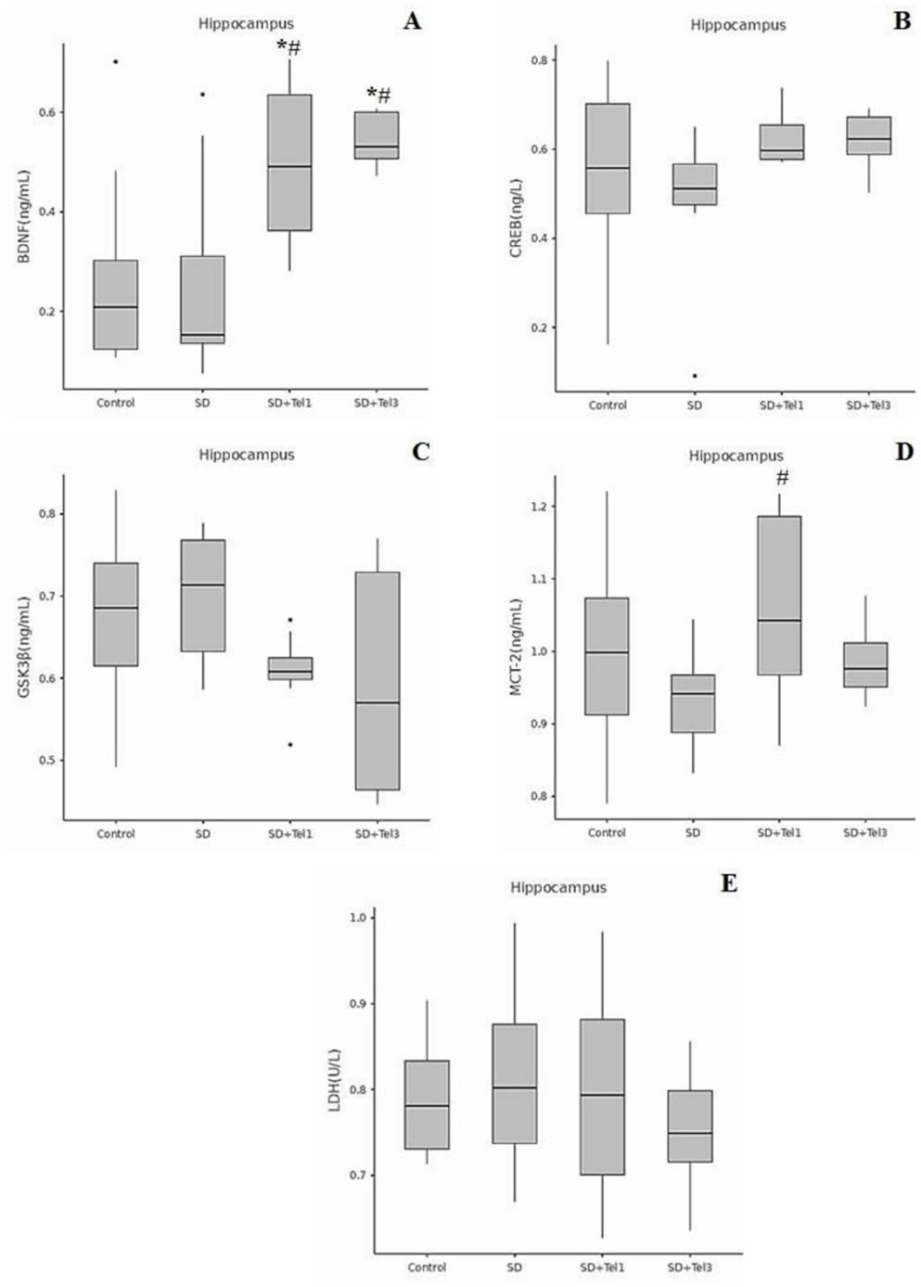
The effect of telmisartan on levels of BDNF, CREB, GSK-3β, MCT2, LDH of rats. **A** BDNF levels in the hippocampus tissue, **B** CREB levels in the hippocampus tissue, **C** GSK-3β levels in the hippocampus tissue, **D** MCT2 levels in the hippocampus tissue, **E** LDH levels in the hippocampus tissue. Group comparisons for numerical variables were conducted using the Kruskal–Wallis test (with post hoc Dunn’s test for pairwise subgroup comparisons when significant differences were detected). The data are expressed as median (min-max). *p < 0.05, significantly different from control group; # *p* < 0.05, significantly different from SD group

**Fig. 4:**
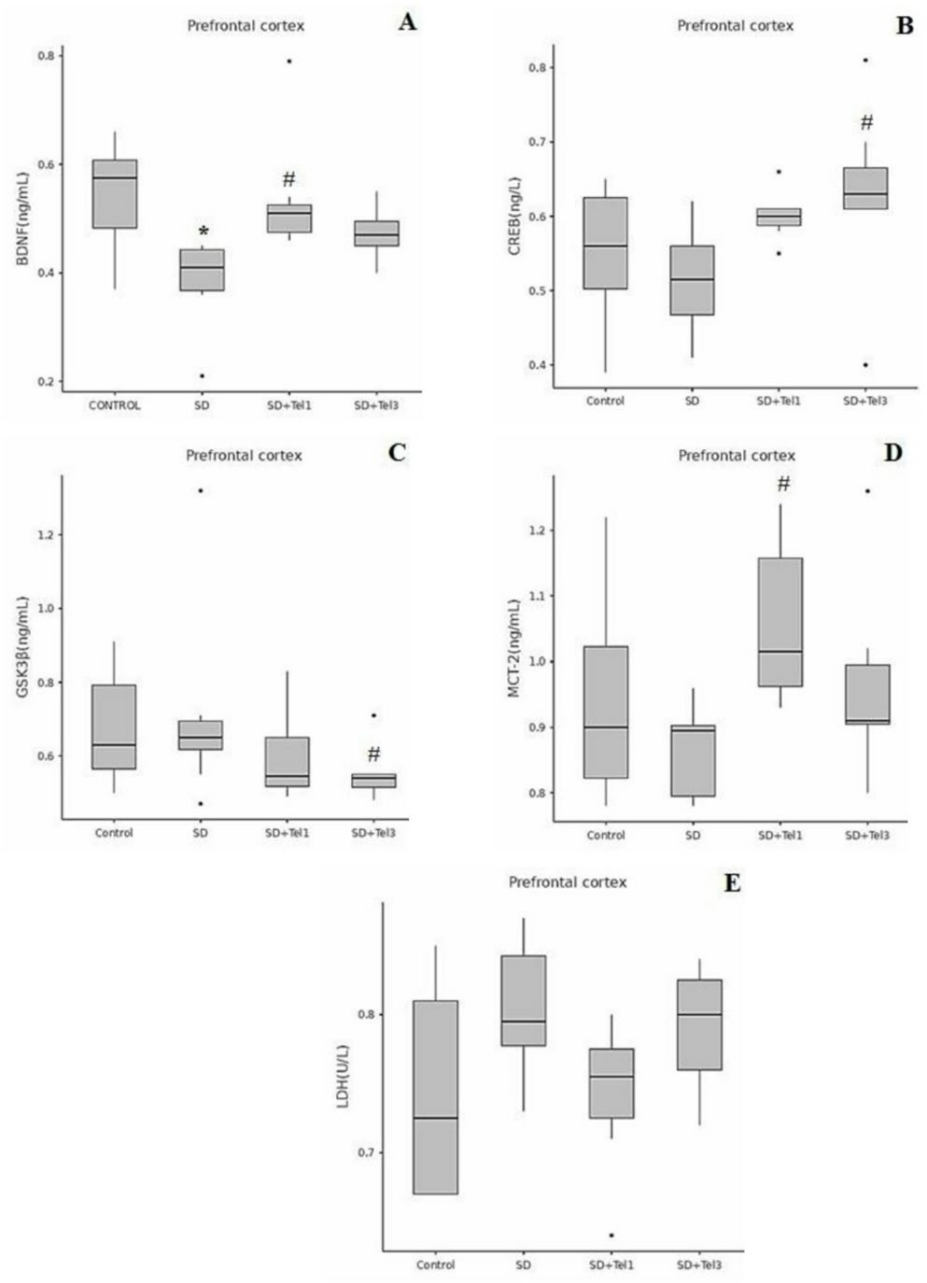
The effect of telmisartan on levels of BDNF, CREB, GSK-3β, MCT2, LDH of rats. **A** BDNF levels in the prefrontal cortex tissue, **B** CREB levels in the prefrontal cortex tissue, **C** GSK-3β levels in the prefrontal cortex tissue, **D** MCT2 levels in the prefrontal cortex tissue, **E** LDH levels in the prefrontal cortex tissue. Group comparisons for numerical variables were conducted using the Kruskal–Wallis test (with post hoc Dunn’s test for pairwise subgroup comparisons when significant differences were detected). The data are expressed as median (min-max). *p < 0.05, significantly different from control group; # *p* < 0.05, significantly different from SD group

Our results showed that in the hippocampus, BDNF levels were significantly increased in both the SD + Tel1 groups compared with Control (H(3) = 10.08, *p* = 0.022, ε² = 0.25) and SD (H(3) = 10.08, *p* = 0.015, ε² = 0.25) groups and SD + Tel3 groups compared with Control (H(3) = 10.08, *p* = 0.017, ε² = 0.25) and SD (H(3) = 10.08, *p* = 0.011, ε² = 0.25) groups, indicating a telmisartan-related recovery. Hippocampal CREB levels did not reach statistical significance (*p* > 0.05); however, both telmisartan groups showed a clear trend toward higher levels relative to SD. In the prefrontal cortex, BDNF was significantly reduced in the SD group compared with controls (H(3) = 14.39, *p* = 0.005, ε² = 0.37), and this decrease was significantly reversed in the SD + Tel1 group (H(3) = 14.39, *p* = 0.008, ε² = 0.37). For CREB, our results demonstrated a significant increase only in the SD + Tel3 group relative to SD (H(3) = 8.33, *p* = 0.033, ε² = 0.21) (Figs. 3 and 4). GSK-3β levels were increased in the hippocampus of SD rats, and telmisartan partially attenuated this increase, although this change did not reach statistical significance (*p* > 0.05), (Fig. 3). In contrast, in the PFC, the SD + Tel3 group exhibited a significant reduction in GSK-3β levels compared with the SD (H(3) = 8.64, *p* = 0.036, ε² = 0.22) group, indicating a region-dependent effect of telmisartan. (Fig. 4) LDH levels did not show significant alterations in either the hippocampus or prefrontal cortex (*p* > 0.05), whereas MCT2 levels were significantly elevated in both the hippocampus (H(3) = 9.53, *p* = 0.015, ε² = 0.30)a nd the prefrontal cortex (H(3) = 10.89, *p* = 0.008, ε² = 0.34) in the SD + Tel1 group compared with the SD group. (*p* < 0.05) (Figs. 3 and 4).

### Influence of REM Sleep Deprivation and Telmisartan Treatment on Plasma CORT Levels and Brain/Body Weight Ratio Levels

**Fig. 5:**
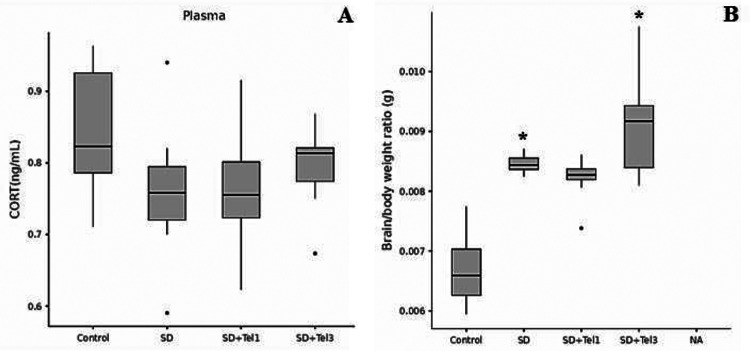
The effect of telmisartan on levels of CORT of rats and brain/body weight ratio levels of rats. **A** CORT levels in plasma, **B** brain/body weight ratio levels. Group comparisons for numerical variables were conducted using the Kruskal–Wallis test (with post hoc Dunn’s test for pairwise subgroup comparisons when significant differences were detected). The data are expressed as median (min–max). *p < 0.05, significantly different from control group

Plasma CORT levels showed no significant change (*p* > 0.05) (Fig. 5A). The brain/body weight ratio was significantly increased in the SD and SD + Tel3 groups compared with the Control group, indicating that REM sleep deprivation induces brain edema. Telmisartan treatment at 3 mg/kg did not prevent this effect (Fig. 5B).

## Discussion

In our study, chronic REM sleep deprivation was associated with region-specific changes in selected neurotrophic, oxidative, and metabolic markers in the hippocampus and prefrontal cortex. In the PFC, BDNF and CREB levels were reduced in the SD group, and telmisartan treatment effectively prevented the decrease in CREB. In contrast, CREB levels in the hippocampus were not significantly affected by sleep deprivation. GSK-3β expression showed an increase following sleep deprivation in the PFC, which was attenuated by telmisartan at a dose of 3 mg/kg. In addition, MCT2 levels were elevated in both regions in the SD + Tel1 group, suggesting a potential enhancement of neuronal energy metabolism. Although LDH activity and plasma CORT levels did not differ significantly among groups, oxidative stress–related parameters showed a favorable profile in telmisartan-treated animals, as reflected by reduced MDA and increased GSH levels. Taken together, these findings indicate that telmisartan may exert neuroprotective effects under conditions of chronic REM sleep deprivation, possibly through modulation of GSK-3β-related signaling, partial normalization of CREB/BDNF pathways in the PFC, support of neuronal energy metabolism, and attenuation of oxidative stress.

Sleep is a vital homeostatic process essential for central nervous system function, energy regulation, and cognitive performance [42]. Chronic sleep deprivation disrupts these processes, leading to impairments in memory and learning, neuronal injury, oxidative stress, and systemic physiological alterations, including changes in body weight, blood glucose, blood pressure, body temperature, and brain/body weight ratio [42]. Sleep deprivation has been widely used as an experimental model to investigate the effects of impaired restorative processes on neuronal function, synaptic plasticity, and energy metabolism [43]. In the present study, we examined the neurobiological consequences of chronic REM sleep deprivation and assessed the potential neuroprotective effects of telmisartan, an angiotensin II receptor blocker with partial PPARγ agonist activity [21], on hippocampal and PFC neurobiology and cognitive function. Our findings offer additional insight into potential mechanisms associated with sleep deprivation–related cognitive impairment and suggest that telmisartan may influence neurotrophic signaling, kinase pathways, oxidative stress markers, and energy metabolism.

Consistent with previous reports [44], CSD was associated with impaired weight gain, as reflected by significantly lower body weight in the SD, SD + Tel1, and SD + Tel3 groups compared with controls. This reduction in body weight is in line with reported alterations in energy metabolism [45] during prolonged wakefulness. However, alterations in body weight observed in REM sleep deprivation models are also frequently associated with stress, hypermetabolism, and changes in feeding behavior. Telmisartan treatment did not appear to normalize body weight, suggesting that its potential neuroprotective effects are not primarily mediated through changes in systemic energy balance. Similarly, sleep-deprived animals showed reduced blood glucose levels, most notably in the SD + Tel1 group, which may be related to telmisartan’s partial PPARγ agonist activity and its known influence on carbohydrate metabolism [21]. Since a stress-control group was not included in the present study, the contribution of nonspecific stress effects to both body weight and glucose alterations cannot be fully ruled out. Accordingly, these results should be interpreted cautiously rather than being ascribed solely to REM sleep loss or telmisartan administration.

Thermoregulatory alterations were observed in sleep-deprived animals, with both SD + Tel1 and SD + Tel3 groups showing reduced body temperature compared to control groups. However, body temperature was measured only at the beginning (day 1) and at the end (week 3) of the experimental protocol, providing a limited snapshot of thermoregulatory status under prolonged REM sleep deprivation and telmisartan treatment. Given that core temperature is highly sensitive to stress exposure, circadian variability, and the effects of anesthesia used during measurement, these reductions should be interpreted cautiously. While the observed changes may suggest a directional effect, they do not allow robust conclusions regarding dynamic or circadian fluctuations. More frequent or continuous temperature monitoring would be required to fully characterize thermoregulation during chronic sleep deprivation. Similarly, blood pressure measurements showed that SD animals exhibited an increase during the first week, while the SD + Tel3 group displayed a partial attenuation of this response, suggesting a possible cardiovascular effect of telmisartan under chronic sleep deprivation [46]. However, given the variability of blood pressure changes across experimental weeks and the absence of a consistent or sustained dose-dependent pattern, these findings should be regarded as descriptive rather than definitive evidence of cardiovascular protection.

The brain-to-body weight ratio was used as a descriptive index reflecting the relationship between brain mass and systemic body weight, which can be influenced by chronic stress and metabolic challenges such as REM sleep deprivation. Therefore, the increase in this ratio observed in the SD and SD + Tel3 groups should not be interpreted as direct evidence of brain edema. Given the marked body weight loss in REM sleep–deprived animals, the elevated ratio may largely reflect reduced body mass, with relative preservation of brain tissue, rather than a true increase in brain volume or clear structural pathology.

BDNF and CREB form a key molecular axis that underlies learning and memory. CREB regulates the transcription of synaptic plasticity-related genes, including BDNF, while BDNF activates TrkB signaling pathways that enhance CREB phosphorylation. This reciprocal interaction strengthens synaptic connections and supports long-term potentiation, thereby facilitating memory consolidation. Disruption of this pathway impairs synaptic plasticity and cognitive performance, whereas interventions that enhance CREB activity or BDNF expression restore neuronal function and learning capacity [47]. These molecular insights provide a framework to understand the region-specific effects of sleep deprivation and the neuroprotective action of telmisartan observed in our study. Among the most consistent findings in the literature is the vulnerability of the hippocampus and PFC to sleep deprivation. Both regions are crucial for memory consolidation and executive function, and their impairment is strongly associated with reduced levels of BDNF and its transcriptional regulator CREB [48]. In our study, hippocampal BDNF levels were significantly elevated in the SD + Tel1 group compared to both control and SD groups, indicating a robust protective effect of telmisartan at this dose. Similarly, BDNF levels in the PFC were significantly decreased in the SD group compared to the control group, but BDNF levels normalized by telmisartan at a dose of 1 mg/kg. These results support previous reports that telmisartan at 1 mg/kg promotes hippocampal BDNF production and extend these findings to the PFC [49]. PFC CREB levels were compared across groups, a statistically significant increase was observed in the SD + Tel3 group compared with the SD group. In the present study, telmisartan treatment was associated with region- and dose-specific changes in BDNF and CREB levels. Given the well-established interplay between BDNF and CREB in synaptic plasticity, these molecular alterations may be relevant to the cognitive improvements observed following telmisartan treatment. Nevertheless, while the study was designed to examine CREB–BDNF-related signalling, the present findings do not allow definitive conclusions regarding the dynamic interactions or causal relationships within this pathway.

As BDNF–CREB activation enhances long-term potentiation and supports hippocampus-dependent memory, subsequent behavioral analyses were performed to examine the impact of sleep deprivation and telmisartan treatment on learning and memory performance. During the four-day training with a hidden platform, all groups showed a gradual decrease in escape latency compared with day one, indicating successful acquisition of the platform location. On the fifth day, memory performance was evaluated by the probe trial, which revealed that animals in the SD and SD + Tel1 groups spent significantly less time in the target quadrant than controls, demonstrating that sleep deprivation impaired memory retention. Previous studies have shown that hippocampus-dependent memory is particularly vulnerable to sleep deprivation, whereas hippocampus-independent memory remains intact [50], supporting the view that hippocampal function is especially affected. Consistent with research showing telmisartan’s positive effects on cognitive function [49], our findings demonstrated that the SD + Tel1 group exhibited a shorter escape latency on the fourth training day compared to the SD group. These findings align with earlier evidence suggesting that NREM sleep enhances synaptic plasticity and performance gains, whereas REM sleep plays a stabilizing role in memory consolidation [51]. Thus, our results support the hypothesis that REM sleep contributes to the stabilization of pre-sleep learning, and suggest that telmisartan may mitigate sleep deprivation–induced hippocampal dysfunction primarily at the level of learning acquisition rather than long-term memory retention. The improvement in learning performance observed following telmisartan treatment at the 1 mg/kg dose may be associated with preserved hippocampal BDNF levels.

Energy metabolism is a critical determinant of neuronal viability under stress conditions. MCTs, and specifically MCT2, are responsible for shuttling lactate from astrocytes to neurons, thereby providing a vital energy substrate [52]. In our study, MCT2 levels in the hippocampus and PFC were significantly increased in SD + Tel1 animals compared to the SD group. However, this increase was not observed consistently across other experimental groups, limiting the interpretation of this finding. Accordingly, the present results do not support definitive conclusions regarding a telmisartan-mediated enhancement of neuronal lactate transport, but instead indicate a dose-specific association. Previous studies have reported reduced MCT2 expression in cerebral ischemia [53] and partial restoration following telmisartan treatment, as well as similar observations in traumatic brain injury models [27]. In this context, our findings suggest a limited and condition-dependent association between angiotensin II inhibition and neuronal energy–related markers under chronic sleep deprivation.

GSK-3β, a serine/threonine kinase highly expressed in the central nervous system, regulates multiple cellular processes, including apoptosis and synaptic function [24]. Our results complement this finding by demonstrating that telmisartan. Hippocampal GSK-3β levels did not differ significantly between groups. In contrast, GSK-3β levels in the PFC were significantly reduced in SD + Tel3 animals relative to SD rats. Importantly, chronic inhibition of GSK-3β has been linked to enhanced neuronal survival, supporting the idea that telmisartan modulates kinase activity in a region-specific manner [24]. Our findings suggest that GSK-3β–related alterations in the PFC under conditions of CSD may be of potential relevance to its role in higher cognitive functions.

Glycogen, as the primary energy reserve in the brain, represents a key component of cerebral energy metabolism and is known to be depleted during prolonged wakefulness and replenished during sleep [54]. In our study, brain glycogen levels were significantly increased in the SD + Tel1 group compared with SD rats, suggesting that telmisartan at this dose is associated with improved glycogen recovery following sleep deprivation. Conversely, SD animals exhibited reduced glycogen levels relative to controls, consistent with evidence indicating that chronic wakefulness leads to cerebral glycogen depletion. Previous research has shown that GSK-3β inhibits glycogen synthase and thereby impairs glycogen synthesis [24]. Although we did not assess GSK-3β phosphorylation status, glycogen synthase activity, or regional glycogen dynamics, our findings indicate dissociable dose-dependent effects of telmisartan, with increased brain glycogen levels observed in the SD + Tel1 group, whereas significant modulation of GSK-3β expression in the prefrontal cortex was detected only in the SD + Tel3 group. These results suggest that telmisartan may influence cerebral energy regulation through mechanisms that are not directly coupled across doses under sleep deprivation. Therefore, these findings do not support a direct causal relationship but suggest that telmisartan may modulate cerebral energy metabolism, including pathways linked to glycogen regulation and GSK-3β–associated signalling, under conditions of sleep deprivation.

LDH catalyzes the reversible conversion of lactate to pyruvate and is commonly used as a general indicator of cellular damage [55]. However, when assessed in tissue homogenates, LDH lacks specificity for neuronal injury and its interpretation is inherently limited. In the present study, LDH levels in both the hippocampus and prefrontal cortex did not differ significantly between experimental groups. Although minor variations in mean values were observed following sleep deprivation and telmisartan treatment, these changes were not statistically significant and do not support conclusions regarding neuronal injury or neuroprotection. In this context, LDH was considered only as a downstream, supportive marker rather than a primary indicator of neuroprotective efficacy. Future studies incorporating region-specific analyses and more sensitive markers of neuronal injury are required to clarify these aspects.

Sleep deprivation has been shown to activate the hypothalamic–pituitary–adrenal (HPA) axis, leading to elevated corticosterone levels during acute stress conditions [56]. However, in chronic sleep deprivation paradigms, the HPA axis may undergo functional adaptation, and several studies have reported significant physiological and neurobiological alterations in the absence of sustained changes in circulating corticosterone levels [10, 57]. In particular, long-term sleep deprivation has been shown to impair synaptic plasticity and increase oxidative stress in the brain without concomitant elevations in plasma corticosterone, suggesting that central nervous system alterations can occur without detectable changes in systemic glucocorticoid output.

Consistent with these reports, we observed no significant differences in plasma corticosterone levels across experimental groups following 21 days of REM sleep deprivation induced by the modified multiple-platform method. This finding may reflect adaptive regulation of the HPA axis during prolonged sleep loss, but alternative explanations related to methodological factors—such as sampling timing, circadian variability, assay sensitivity, and the absence of a dedicated stress-control group cannot be excluded. Therefore, the CORT data should be interpreted cautiously, and no definitive conclusions regarding HPA-axis adaptation can be drawn. Importantly, these findings indicate that the protective effects observed in other outcome measures are unlikely to be mediated by changes in circulating corticosterone and may instead involve mechanisms operating independently of overt systemic glucocorticoid modulation.

Oxidative stress is recognized as an important contributor to neuronal alterations associated with sleep deprivation. MDA, a lipid peroxidation product, was assessed as an index of oxidative damage, while GSH was measured as a key component of endogenous antioxidant defense. In the present study, brain MDA levels were significantly reduced and GSH levels were significantly increased only in the SD + Tel3 group compared with the SD group, indicating a dose-dependent and condition-specific effect of telmisartan rather than a generalized antioxidant action. In the absence of additional oxidative stress markers or enzymatic activity measurements, such as superoxide dismutase or catalase activity, these findings should be interpreted cautiously. Accordingly, the observed changes in MDA and GSH are best viewed as reflecting an alteration in redox balance associated with high-dose telmisartan treatment, rather than definitive evidence of neuroprotection. Overall, the present data support a modulatory role of telmisartan on oxidative processes under REM sleep deprivation conditions, without allowing firm conclusions regarding its protective efficacy on oxidative stress–related neuronal injury.

Nitric oxide (NO) is an essential signaling molecule involved in cerebral blood flow regulation, neuronal activity, and inflammatory processes [58]. Owing to its short half-life, NO is commonly assessed via its stable metabolites, nitrate and nitrite [59]. In the present study, brain nitrate levels were significantly elevated in both the SD and SD + Tel3 groups compared with controls, and neither 1 mg/kg nor 3 mg/kg telmisartan reduced these levels. This finding indicates that REM sleep deprivation is associated with increased nitrosative stress that persists despite telmisartan treatment.

Moreover, the similar elevation of nitrate levels in both SD and SD + Tel3 groups suggests that nitrosative pathways are not uniformly suppressed by telmisartan under REM sleep deprivation conditions and may be regulated independently from other redox-related processes. In line with previous reports, these findings imply that telmisartan may have limited efficacy on nitrosative pathways when administered as monotherapy [60], underscoring the need for caution in attributing antioxidant or antinitrosative effects based solely on nitrate measurements.

### Limitations of the Study

While the present study provides new insights into the effects of chronic REM sleep deprivation and telmisartan treatment on cognitive, molecular, and metabolic parameters, several limitations should be acknowledged. First, biochemical and oxidative stress markers were assessed using non–region-specific brain tissue, which may have masked region-dependent alterations known to occur during REM sleep deprivation and limited direct comparison with region-specific hippocampal and prefrontal cortex analyses.

Second, the absence of a stress-matched or large-platform control group in the modified multiple-platform paradigm restricts the ability to fully dissociate REM-specific effects from nonspecific stress-related influences. Third, oxidative stress assessment relied on the thiobarbituric acid–based MDA assay, which has limited specificity for lipid peroxidation, and additional enzymatic or region-specific redox markers were not evaluated.

Fourth, body temperature was measured only at two time points, precluding assessment of temporal or circadian variability in thermoregulation. Finally, direct measures of cerebral edema, such as tissue water content or volumetric analyses, were not performed; therefore, changes in the brain-to-body weight ratio should be interpreted cautiously, as they may primarily reflect REM sleep deprivation–associated body weight loss rather than structural brain alterations.

Future studies incorporating stress-control groups, region-specific sampling, longitudinal physiological monitoring, and more selective molecular approaches will be necessary to further clarify the mechanisms underlying these findings.

## Conclusion

The present study demonstrates that chronic sleep deprivation disrupts multiple neurobiological pathways, including neurotrophic signaling, kinase regulation, oxidative stress balance, and energy metabolism. Telmisartan treatment, particularly at doses of 1 mg/kg and 3 mg/kg, was associated with partial attenuation of several of these changes, including region-specific modulation of BDNF levels, enhancement of antioxidant-related parameters, increased MCT2 expression, and alterations in glycogen metabolism. In contrast, telmisartan did not affect plasma corticosterone or brain nitrate levels, suggesting that its effects are unlikely to be mediated by systemic stress hormone regulation or global nitric oxide–related pathways. Taken together, these findings suggest that telmisartan may exert neuroprotective effects under conditions of sleep deprivation by influencing trophic and metabolic signalling processes in the hippocampus and PFC, regions critically involved in learning and memory. While the present results are encouraging, further studies are needed to better delineate dose-dependent effects, assess potential combination therapies, and evaluate the relevance of these findings in translational and clinical contexts of chronic sleep loss.

## Data Availability

The datasets generated during the current study are available from the corresponding author on reasonable request.
